# Supplementary material for: The impact of FASTQ and alignment read order on structural variant calling from long-read sequencing data
Source: PeerJ. 2024 Mar 15;12:e17101. doi: 10.7717/peerj.17101 (PMC10946394; doi:10.7717/peerj.17101)
Supplement: Supplemental Information 1 — 1. SV call sets were generated from Minimap2 aligned and SAMtools sorted BAM files (20X depth). 2. Values represent the global mean of the Jaccard distances between the original and randomized FASTQ files for each strain or ecotype. Values outside parentheses describe the VCF level differences. Values inside parentheses describe the coordinate level differences. 3. The identification of interspersed duplications was only supported by SVIM. [file peerj-12-17101-s001.docx]

Table S1: Overall Jaccard distances for each SV type in *C. elegans* and *A. thaliana*.

| Species | Caller^1^ | BND^2^ | DEL^2^ | DUP^2^ | DUP:I^2,3^ | INS^2^ | INV^2^ | Total^2^ |
| --- | --- | --- | --- | --- | --- | --- | --- | --- |
| *C. elegans* | pbsv | 0.077 (0.004) | 0.019 (0.003) | 0.040 (0.022) | NA | 0.021 (0.012) | 0.011 (0.000) | 0.022 (0.009) |
|  | sniffles | 0.000 (0.000) | 0.000 (0.000) | 0.000 (0.000) | NA | 0.007 (0.007) | 0.000 (0.000) | 0.004 (0.004) |
|  | svim | 0.000 (0.000) | 0.002 (0.001) | 0.000 (0.000) | 0.000 (0.000) | 0.002 (0.002) | 0.000 (0.000) | 0.002 (0.002) |
| *A. thaliana* | pbsv | 0.105 (0.007) | 0.008 (0.001) | 0.075 (0.051) | NA | 0.021 (0.012) | 0.107 (0.000) | 0.017 (0.007) |
|  | sniffles | 0.000 (0.000) | 0.000 (0.000) | 0.000 (0.000) | NA | 0.012 (0.012) | 0.000 (0.000) | 0.006 (0.006) |
|  | svim | 0.000 (0.000) | 0.005 (0.002) | 0.019 (0.017) | 0.000 (0.000) | 0.005 (0.004) | 0.003 (0.000) | 0.005 (0.004) |

1. SV call sets were generated from Minimap2 aligned and SAMtools sorted BAM files (20X depth).
2. Values represent the global mean of the Jaccard distances between the original and randomized FASTQ files for each strain or ecotype. Values outside parentheses describe the VCF level differences. Values inside parentheses describe the coordinate level differences.
3. The identification of interspersed duplications was only supported by SVIM.
