# Supplementary figures and images for: The impact of FASTQ and alignment read order on structural variant calling from long-read sequencing data
Source: PeerJ. 2024 Mar 15;12:e17101. doi: 10.7717/peerj.17101 (PMC10946394; doi:10.7717/peerj.17101)

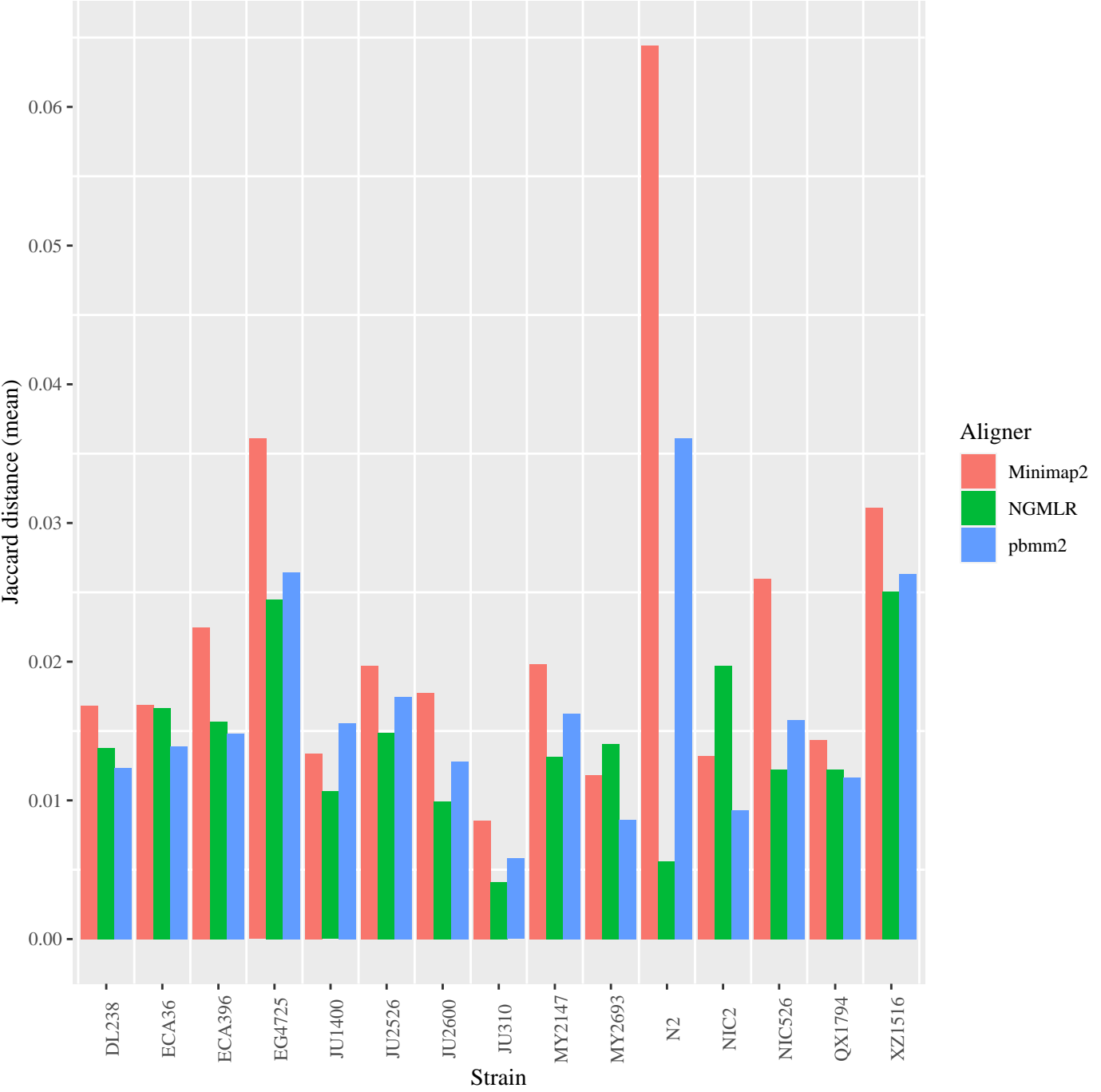

Supplement: Supplemental Information 6 — For each strain, five files with randomized read orders were created from the original FASTQ file. Each FASTQ file was subsampled to ensure that the alignment depths of each aligner were 20X and the BAM files were sorted using SAMtools. The Jaccard distances describe the proportion of predictions that were in disagreement between the call sets generated from the original and randomized FASTQ files. [file peerj-12-17101-s006.pdf]

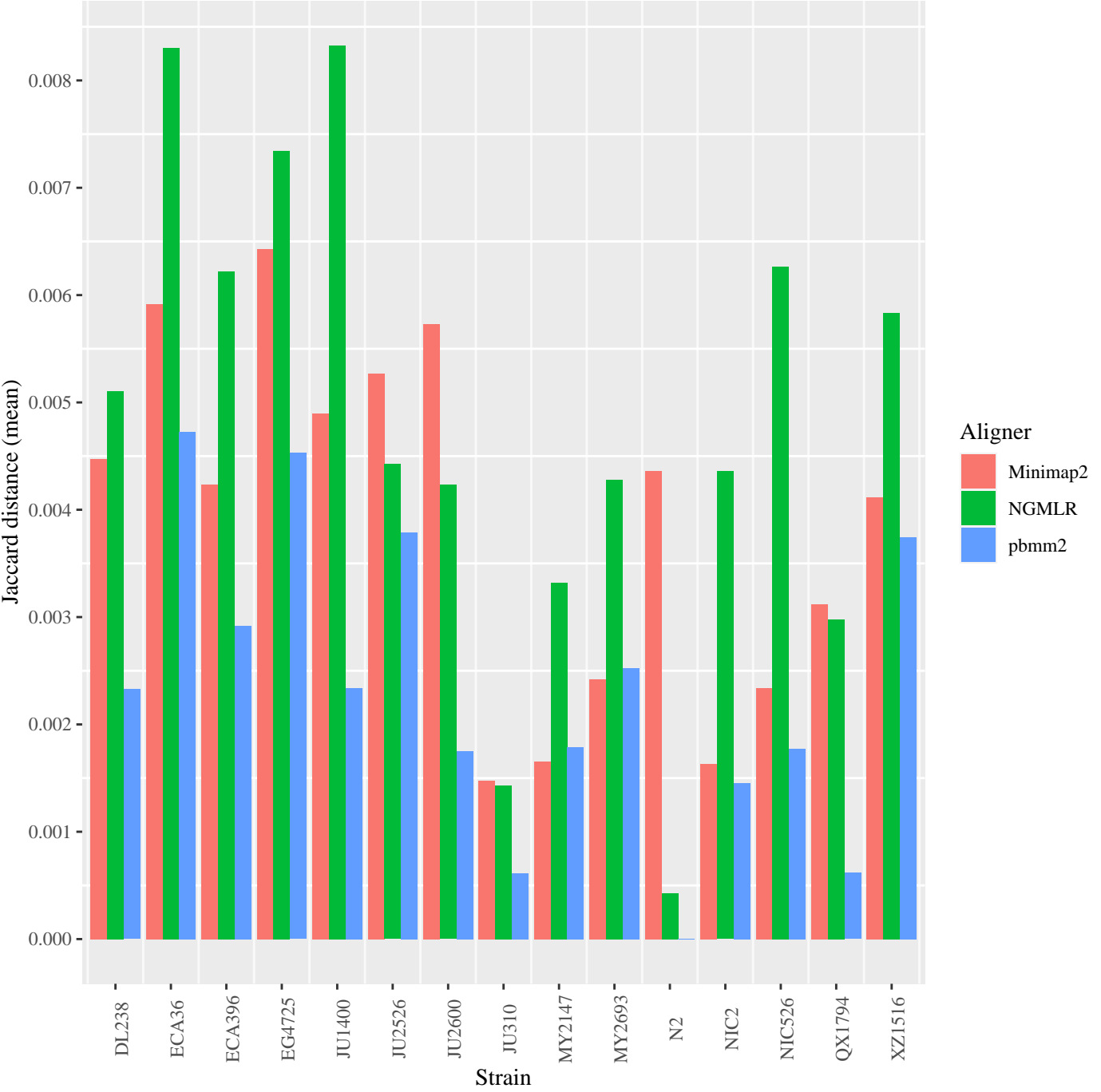

Supplement: Supplemental Information 7 — For each strain, five files with randomized read orders were created from the original FASTQ file. Each FASTQ file was subsampled to ensure that the alignment depths of each aligner were 20X and the BAM files were sorted using SAMtools. The Jaccard distances describe the proportion of predictions that were in disagreement between the call sets generated from the original and randomized FASTQ files. [file peerj-12-17101-s007.pdf]

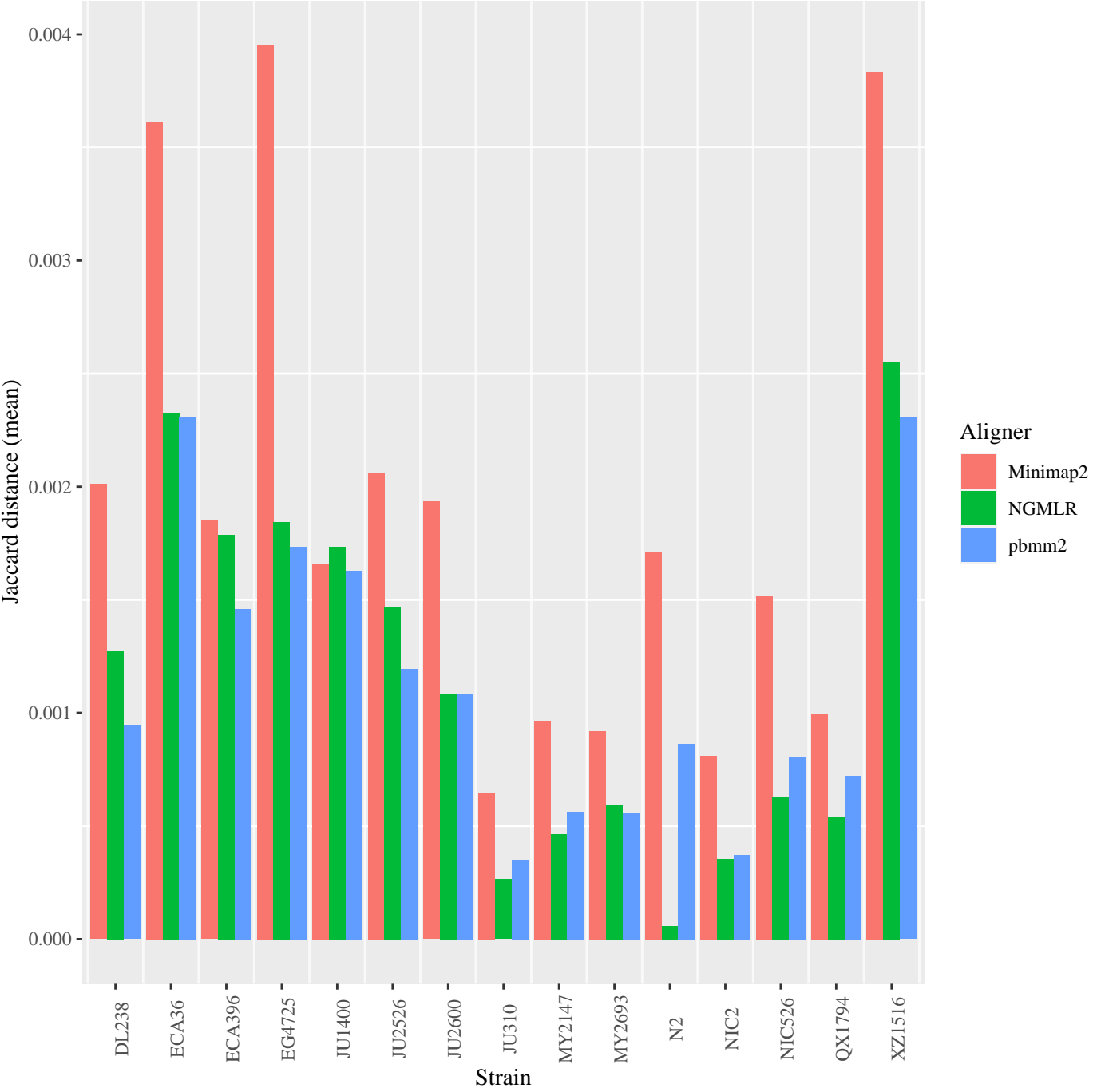

Supplement: Supplemental Information 8 — For each strain, five files with randomized read orders were created from the original FASTQ file. Each FASTQ file was subsampled to ensure that the alignment depths of each aligner were 20X and the BAM files were sorted using SAMtools. The Jaccard distances describe the proportion of predictions that were in disagreement between the call sets generated from the original and randomized FASTQ files. [file peerj-12-17101-s008.pdf]

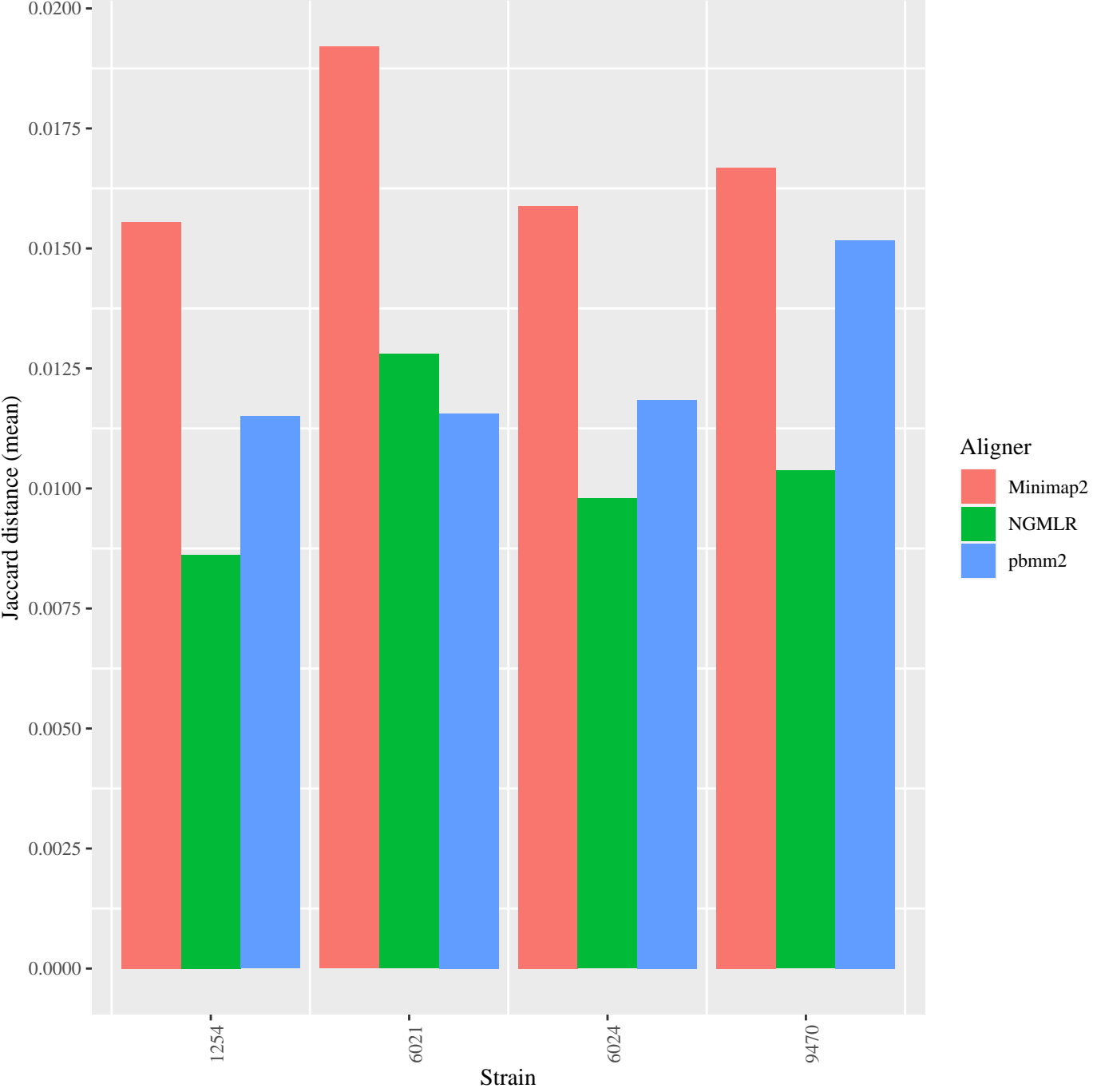

Supplement: Supplemental Information 9 — For each ecotype, five files with randomized read orders were created from the original FASTQ file. Each FASTQ file was subsampled to ensure that the alignment depths of each aligner were 20X and the BAM files were sorted using SAMtools. The Jaccard distances describe the proportion of predictions that were in disagreement between the call sets generated from the original and randomized FASTQ files. [file peerj-12-17101-s009.pdf]

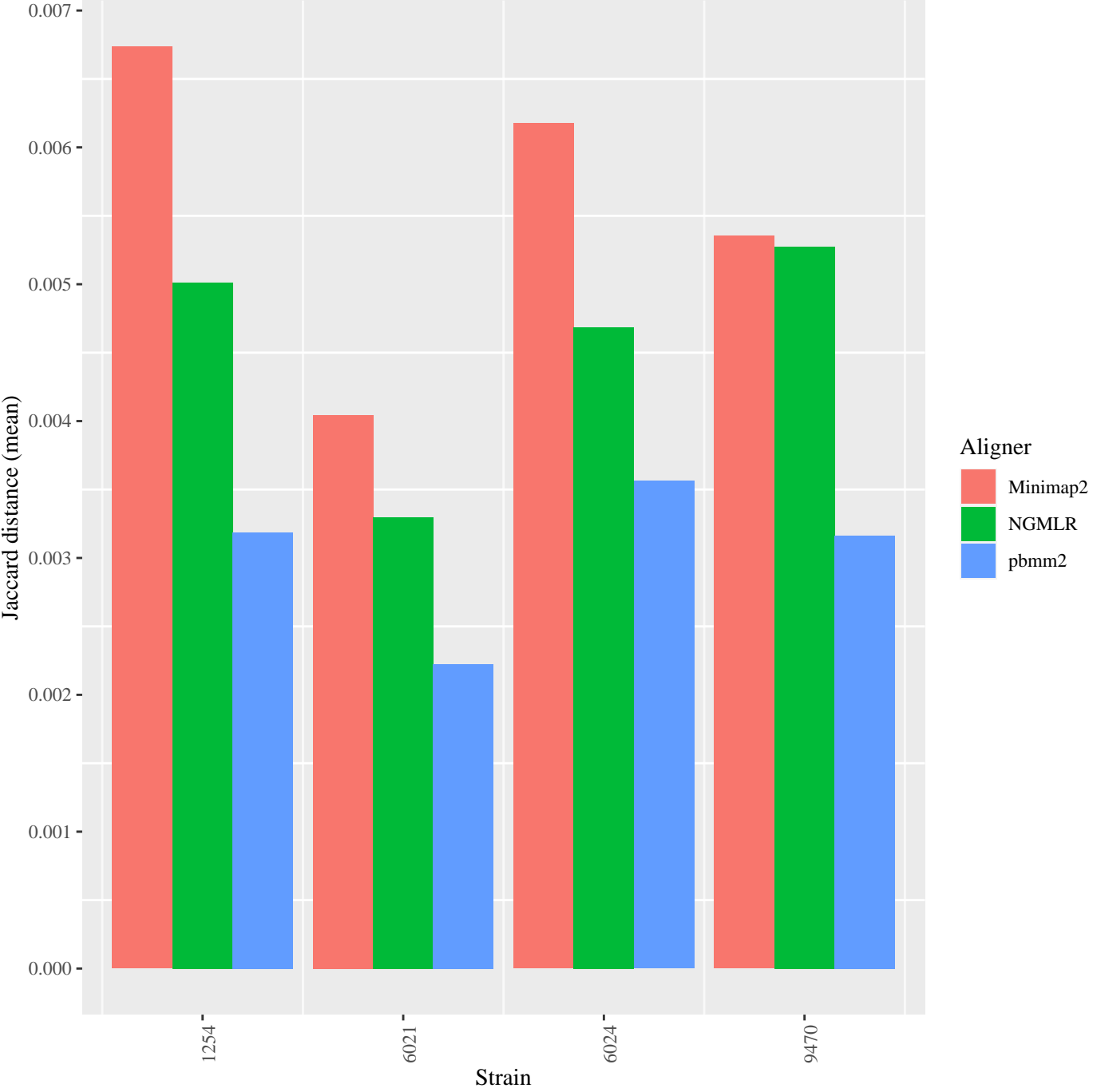

Supplement: Supplemental Information 10 — For each ecotype, five files with randomized read orders were created from the original FASTQ file. Each FASTQ file was subsampled to ensure that the alignment depths of each aligner were 20X and the BAM files were sorted using SAMtools. The Jaccard distances describe the proportion of predictions that were in disagreement between the call sets generated from the original and randomized FASTQ files. [file peerj-12-17101-s010.pdf]

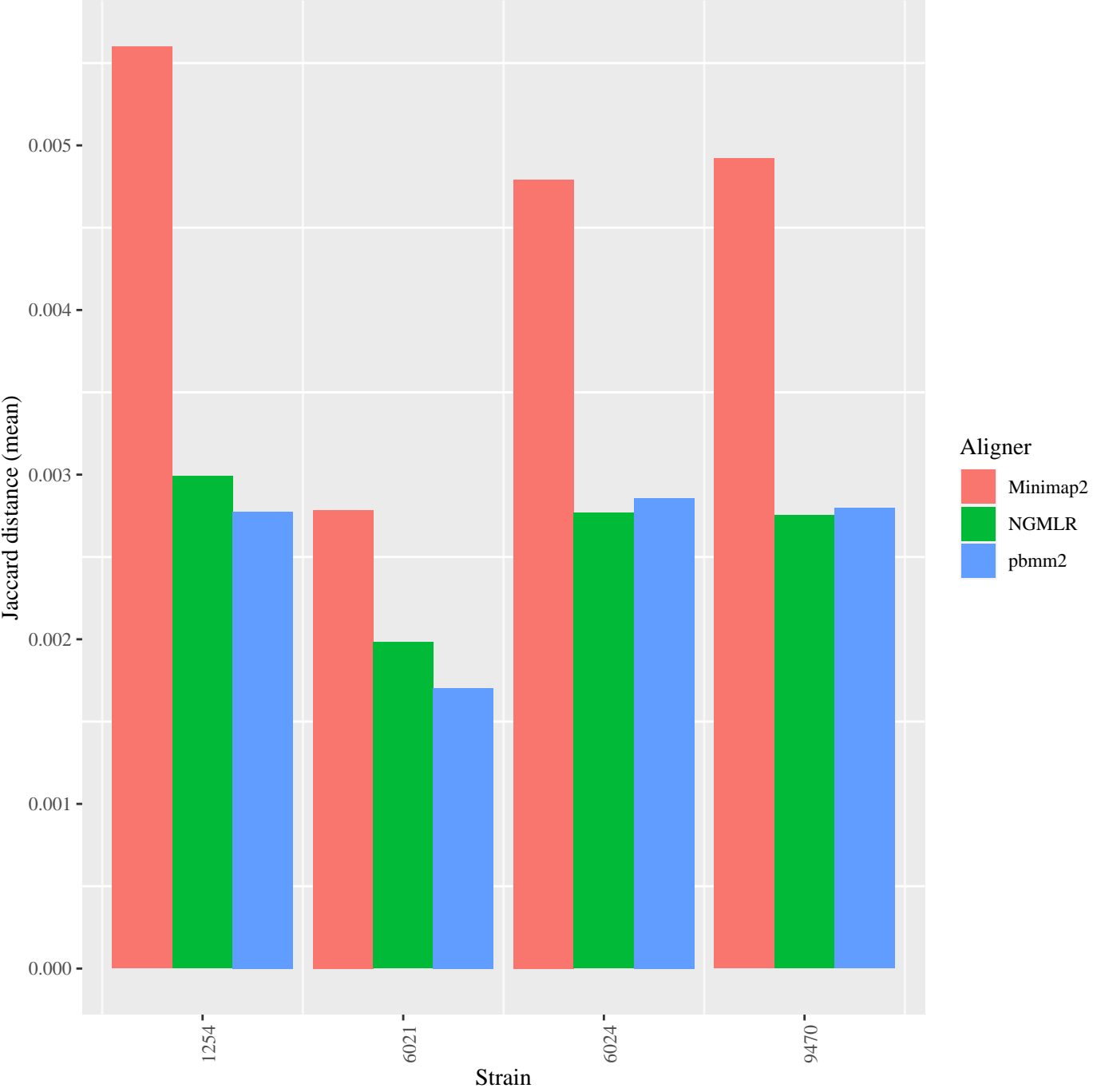

Supplement: Supplemental Information 11 — For each ecotype, five files with randomized read orders were created from the original FASTQ file. Each FASTQ file was subsampled to ensure that the alignment depths of each aligner were 20X and the BAM files were sorted using SAMtools. The Jaccard distances describe the proportion of predictions that were in disagreement between the call sets generated from the original and randomized FASTQ files. [file peerj-12-17101-s011.pdf]

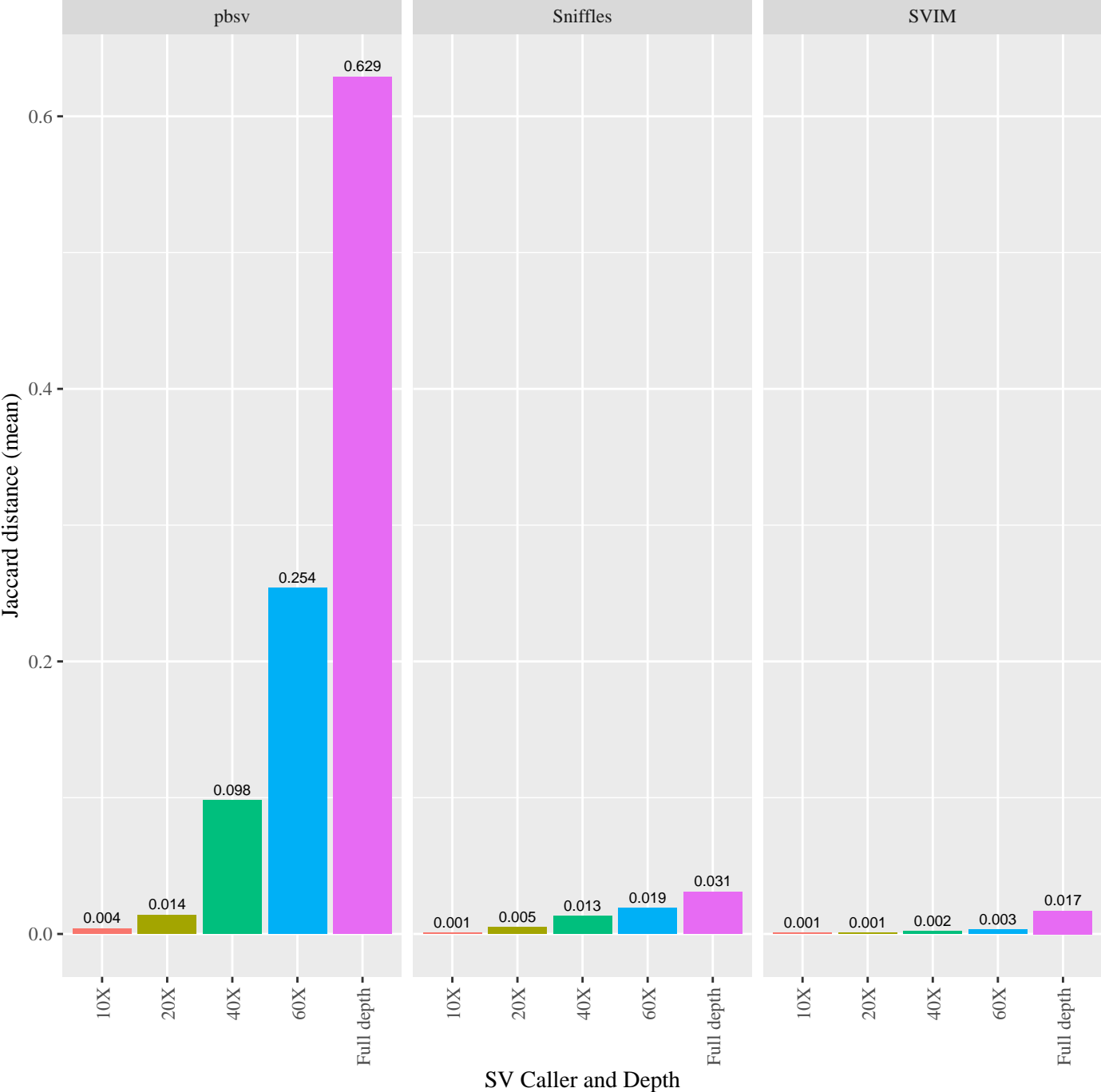

Supplement: Supplemental Information 12 — For each strain, five files with randomized read orders were created from the original FASTQ file and subsampled to 10X, 20X, 40X, and 60X depth. The subsampled and full depth FASTQ files were aligned in NGMLR and, subsequently, sorted using SAMtools. The Jaccard distances describe the proportion of predictions that were in disagreement between the call sets generated from the original and randomized FASTQ files. [file peerj-12-17101-s012.pdf]

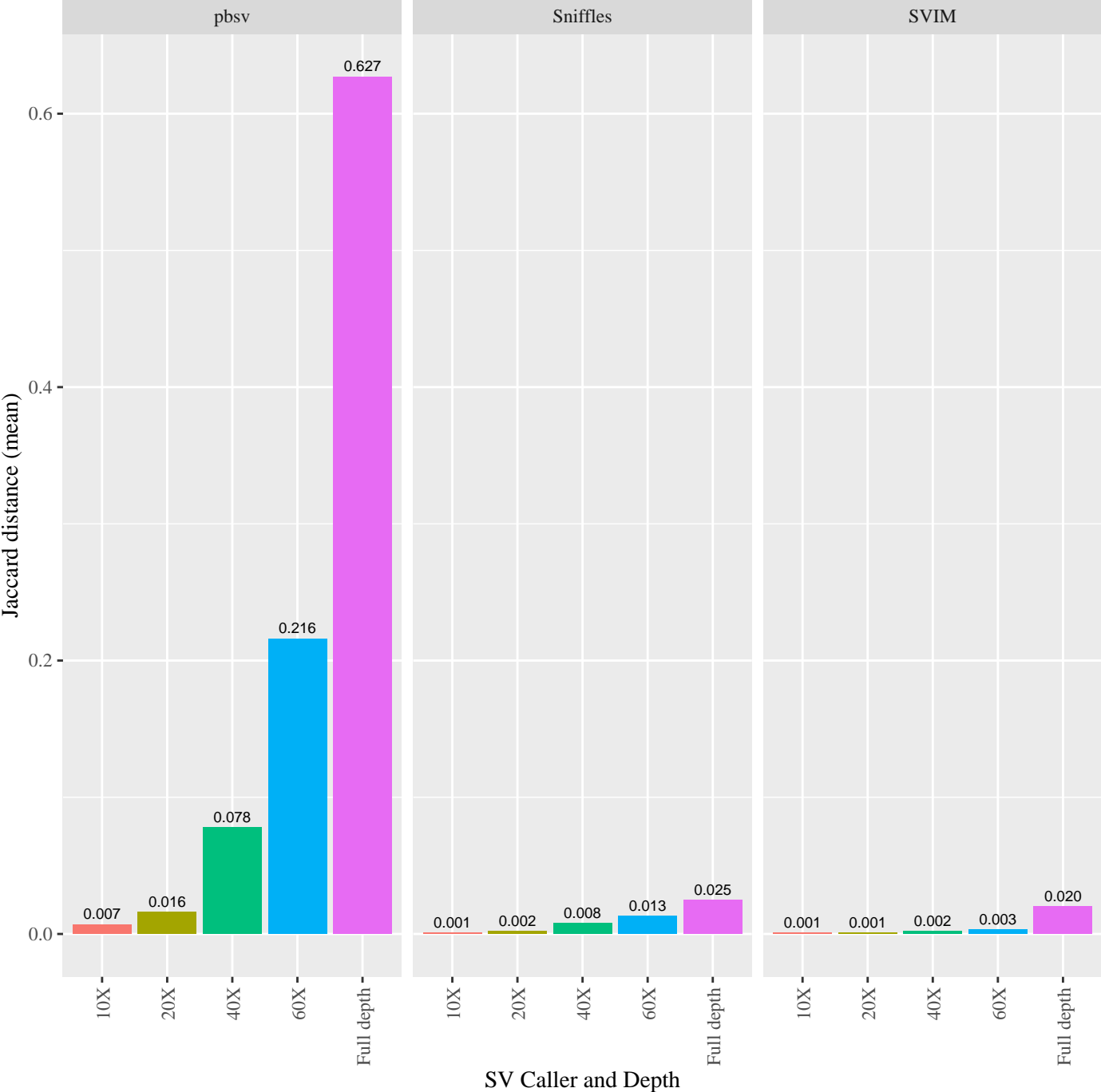

Supplement: Supplemental Information 13 — For each strain, five files with randomized read orders were created from the original FASTQ file and subsampled to 10X, 20X, 40X, and 60X depth. The subsampled and full depth FASTQ files were aligned in pbmm2 and, subsequently, sorted using SAMtools. The Jaccard distances describe the proportion of predictions that were in disagreement between the call sets generated from the original and randomized FASTQ files. [file peerj-12-17101-s013.pdf]
